# Identification of phytase producing bacteria from acidifying *Tithonia diversifolia*: Potential for ruminant feed development

**DOI:** 10.1016/j.sjbs.2024.104006

**Published:** 2024-05-08

**Authors:** Roni Pazla, Gusri Yanti, Novirman Jamarun, Mardiati Zain, Hera Dwi Triani, Ezi Masdia Putri, Anifah Srifani

**Affiliations:** a Department of Animal Nutrition and Feed Technology, Faculty of Animal Science, Universitas Andalas, Limau Manis, Padang 25163, Indonesia; b Department of Agricultural Extension, Faculty of Social, Science and Education, Prima Nusantara Bukittinggi University, Bukittinggi 26122, Indonesia; c Research Center for Animal Husbandry, Research Organization for Agriculture and Food, National Research and Innovation Agency (BRIN) Indonesia, Jl. Raya Jakarta-Bogor, Cibinong 16915, Indonesia; d Doctoral Student of Animal Nutrition and Feed Technology, Faculty of Animal Science, Universitas Andalas, Limau Manis, Padang 25163, Indonesia

**Keywords:** Bacteria, Phytase, *Tithonia diversifolia*, Animal feed

## Abstract

Phytate content in feed ingredients can negatively impact digestibility and palatability. To address this issue, it is necessary to study microbes capable of breaking down phytate content. This study aimed to isolate and characterize phytase-producing bacteria from decaying materials rich in phytic acid. The research was conducted in several stages. The first stage involved isolating phytase-producing bacteria from the acidification of *Tithonia diversifolia* using growth media containing Na-phytate. Bacterial isolates that produced clear zones were then tested for their activity and ability to produce several enzymes, specifically phytase, cellulase, and protease. The next step was to test the morphological characteristics of the bacterial isolate. The final stage of bacterial identification consisted of DNA isolation, followed by PCR amplification of the 16S rRNA gene, DNA sequence homology analysis, and construction of a phylogenetic tree. Based on research, three isolates were found to produce clear phytase zones: isolates R5 (20.3 mm), R7 (16.1 mm) and R8 (31.7 mm). All isolates were able to produce the enzymes phytase (5.45–6.54 U/ml), cellulase (2.60–2.92 U/ml), and protease (22.2–23.4 U/ml). Metagenomic testing identified isolate R7 and R8 as *Alcaligenes faecalis* and isolate R5 as *Achromobacter xylosoxidans*. The isolation and characterization of phytase-producing bacteria from *Tithonia diversifolia* acidification resulted in the identification of two promising candidates that can be applied as sources of phytase producers. Phytase-producing bacteria can be utilized to improve digestibility and palatability in animal feed.

## 1 Introduction

Myo-inositol 1,2,3,4,5,6-hexakisdihydrogenphosphate, commonly referred to as phytic acid, and the mixed cation salt of phytic acid are a class of phosphorus (P) in organic form that are prevalent in many natural ingredients, particularly in nuts, seeds, and oilseed crops, which are important sources of nutrients for animals. This plant contains phytic acid, an essential component in its salt form known as phytate, which is an anhydrous storage type for more than 80 % of the total phosphorus found in cereals and nuts. In terrestrial ecosystems, phytic acid is produced by plants and formed within seeds during the ripening process, and is considered the primary storage form of phosphorus in grains (Turner et al., 2011), as well as myo-inositol (an important growth factor).

Plants hold considerable potential as a source of animal feed, particularly for ruminants. However, the presence of high levels of phytic acid can restrict their utilization in livestock diets due to the animals' limited ability to break down this compound. A noteworthy example is *Tithonia diversifolia*, commonly known as the “Mexican Sunflower,” which is widely distributed in tropical and subtropical regions. This plant has garnered interest as a potential feed source for ruminants owing to its rich nutrient profile, which includes proteins, fibers, and minerals. Despite these beneficial qualities, the presence of phytates in *Tithonia diversifolia* can impede nutrient digestibility and absorption in ruminants, potentially reducing its effectiveness as a livestock feed (Pazla et al., 2021a, Pazla et al., 2021b, Jamarun et al., 2023).

From these considerations, efforts are needed to improve the use of *Tithonia diversifolia* for animal feed, one of which is to convert phytate into compounds that are beneficial for the digestibility of ruminant feed. One way to accomplish this is by identifying phytase-producing bacteria derived from the acidification of *Tithonia diversifolia*, with the hope of increasing the palatability and digestibility of ruminant feed. The study of the phytase enzyme receives great interest in biotechnology applications, especially for reducing phytate in feed and food (Vohra and Satyanarayana, 2003, Ajith et al., 2018).

Phytase-producing bacteria are a group of microorganisms with the ability to hydrolyze phytate, releasing inorganic phosphate that is readily available. During the acidification process of *Tithonia diversifolia*, these bacteria play a role in reducing the phytate content in animal feed. This reduction in phytate is critical, as it enhances the digestibility of feed and improves the efficiency of nutrient utilization by farm animals.

A limited group of bacterial species with the ability to produce phytase has been identified, including strains such as Escherichia coli, anaerobic rumen bacteria, and various species within the genus Pseudomonas. Notable examples of these phytase-producing organisms include Megasphaera elsdenii, Prevotella spp., Selemonas ruminantium, Raoultella spp., and Mitsuokella multiacidus (Bikash et al., 2017). In addition, certain bacteria are classified as non-specialist phytase producers, yet they exhibit optimal efficiency in hydrolyzing phytate into phosphoric acid. This group includes bacteria like Achromobacter, which has potential applications in the formulation of animal feed, especially for ruminants (Bikash et al., 2017). The effectiveness of these non-specialist phytase-producing bacteria in breaking down phytate into phosphoric acid and other related compounds is influenced by specific substrate and growth conditions. For example, Alcaligenes spp. demonstrate optimal growth at pH ranges between 7.0 and 8.0, suggesting their potential use in the production of fish feed (Kumar et al., 2012).

Several previous studies have successfully isolated phytase-producing bacteria from various sources. Nagar et al. (2021) discovered 6 phytase-producing bacterial isolates obtained from soil samples collected from multiple locations, exhibiting halo zones on phytase spesific media ranging from 8 to 14 mm. Based on identification through 16SrRNA gene analysis, two of these bacteria were identified as *Enterobacter cloacae*, two as *Bacillus megaterium*, while others were identified as *Paenibacillus* sp. and *Klebsiella variicola*. Similarly, Nezhad et al. (2023) isolated eleven phytase-producing microorganisms from a more diverse range of sources, including the bottom of a lake, cow stool, compost, decaying corn fruit, soil, and pineapple. Sequencing analysis of 16S rDNA and nuclear ribosomal transcribed spacer (ITS) genes revealed that six of the eleven isolates belonged to the *Acinetobacter* genus, with two isolates affiliated with the *Enterobacter* genus. Additionally, one isolate was associated with the *Pseudomonas* genus, one with the *Escherichia* genus, and another with the *Saccharomyces* genus.

The study on the identification of phytase-producing bacteria in *Tithonia diversifolia* can generate new knowledge about the phytase bacteria present during the acidification of *Tithonia diversifolia*. This research can offer insights into the diversity of phytase-producing microbes and the potential use of these microbes as feed supplements or additives to enhance the quality of ruminant feed.

## 2 Materials and methods

### 2.1 Ethical approval

This research did not need approval in ethics because it did not use animals as the material of study.

### 2.2 Study period and location

The study was carried out between January and May 2023. Tithonia plants were sourced from the city of Padang Panjang, located in West Sumatra, Indonesia. This city is positioned at an altitude ranging from 650 to 850 m above sea level. The average temperature in this region varies between 26.1 °C and 28.1 °C, and the annual rainfall averages around 3,295 mm. The plants are cultivated in Andisol soil. Geographically, Padang Panjang is situated between 100°20′ and 100°27′ East Longitude, and 0°27′ and 0°30′ South Latitude.

### 2.3 Research design

The research was conducted in several stages. The first stage involved isolating phytase-producing bacteria from the acidification of *Tithonia diversifolia* using growth media containing Na-phytate. A series of tests were carried out to select isolates capable of producing phytase, including clear zone and phytase enzyme activity assays. The subsequent step involved DNA isolation, followed by PCR amplification of the 16SrRNA gene and DNA sequence homology analysis. The final step was to construct a phylogenetic tree to determine the evolutionary relationship between the isolates.

### 2.4 Screening of phytase producing bacteria

Bacterial isolates were purified in Nutrient Broth and then subjected to centrifugation at 12,000 rpm for a duration of 5 min. To identify phytase-producing bacteria, a selective medium with the following composition was used: 2 g of glucose, 0.4 g of sodium phytate, 0.2 g of calcium chloride, 0.5 g of ammonium nitrate, 0.05 g of potassium chloride, 0.05 g of magnesium sulfate, 0.01 g of iron sulfate, 0.01 g of manganese sulfate, 1.5 g of Bacto agar, and 100 ml of distilled water. The medium was dispensed into a Petri dish, and a blank paper disc was soaked in the supernatant of the isolate. This paper disc was then placed on the phytase detection medium and incubated at 37 degrees Celsius for 24 h. After the incubation period, a clear zone around the paper disc indicated the presence of phytase-producing bacteria.

### 2.5 Enzyme activities

**Phytase enzyme activity**

To measure phytase enzyme activity, 0.15 ml of enzyme was added to 0.6 ml of Tris-HCl buffer (0.1 M) containing Ca-phytate and CaCl_2_, followed by the addition of 0.75 ml of a 5 % trichloroacetic acid (TCA) solution. This mixture was then incubated at 37 °C for 30 min. After incubation, 1.5 ml of the enzyme solution was mixed with a molybdate color reagent and homogenized. The absorbance of the resulting color was then measured at 700 nm using a spectrophotometer, with a blank as the reference point. Phytase activity measurements were carried out according to Kim and Lei (2005), and the results were calculated using the following formula:

$$PA\;=\;\frac{y-b}{a}$$

where:

• PA represents phytase activity,
• y is sample absorbance,
• a is the “a” value from the regression curve Y = a + bx,
• b is the “b” value from the same regression curve.

**Cellulase enzyme activity**

To measure cellulase enzyme activity, the protocol outlined by Jennifer and Thirunelakandan (2015) was followed. A total of 1 ml of enzyme supernatant was added to 1 ml of an extract consisting of 0.5 ml of carboxymethyl cellulose (CMC) and 10 ml of acetate buffer. This mixture was then incubated at 40 degrees Celsius in a water bath for 30 min. Following incubation, 1 ml of the solution was combined with 1 ml of Nelson's reagent (Nelson AB) and heated in boiling water for 20 min. After the mixture cooled, 1 ml of phosphomolybdate and 7 ml of distilled water were added. Absorbance readings were taken at 575 nm using a spectrophotometer to determine the level of cellulase enzyme activity.

**Protease enzyme activity**

To assess the proteolytic activity of crude enzyme extracts, the procedure outlined by Cupp and Enyard (2008) was employed. A 0.65 % casein substrate was added to 1 ml of the crude enzyme extract, and the mixture was incubated at 37 degrees Celsius for 10 min. The enzymatic reaction was halted by adding 5 ml of 110 mM trichloroacetic acid (TCA) reagent, after which the mixture was incubated for an additional 30 min. Subsequently, 2 ml of the reaction mixture was subjected to centrifugation at 10,000 rpm for 10 min to separate the filtrate. The filtrate was then combined with 5 ml of sodium carbonate (Na_2_CO_3_) and 1 ml of Folin-Ciocalteu reagent, followed by a 30-minute incubation. The absorbance was measured at a wavelength of 660 nm.

### 2.6 Morphological characteristics of phytase-producing bacteria

Phytase-producing bacteria were identified through microscopic examination, focusing on their size, shape, and color. Additionally, Gram staining tests were conducted to determine the Gram status of the isolates. Macroscopically, the bacterial colonies displayed a cream color with an average diameter of approximately 2 mm. Upon microscopic inspection, the isolates exhibited a rod-like or bacillus-shaped morphology.

### 2.7 Identification of isolates using gen 16SrRNA

**Genomic DNA isolation**

Approximately 10 ml of liquid bacterial culture was harvested. The cell pellet was collected and mixed with 200 μl of lysis solution, then vigorously vortexed to ensure proper lysis. The addition of 20 μl of proteinase K was followed by vortexing, and the microtubes were incubated at 56 °C for 30 min, with periodic vortexing every 10 min for 5 s to maintain thorough mixing. Following incubation, 20 μl of RNase A solution was added to the microtube, and it was vortexed for 5 s, followed by an additional incubation at room temperature for 10 min. After the incubation, 400 μl of cold 50 % ethanol was added to the microtube, and the mixture was vortexed for 5 s. The solution was then transferred to a purification column and centrifuged at 8,000^x^g for 1 min. The collection tube, containing the flow-through, was discarded, while the purification column was transferred to a new collection tube. Subsequently, 500 μl of Wash Buffer I was added to the purification column, and it was centrifuged at 8,000^x^g for 1 min. Again, the collection tube was discarded, and the purification column was moved to another clean collection tube. Following this, 500 μl of Wash Buffer II was added to the purification column, and centrifugation was carried out at 10,000 xg for 3 min. The purification column was then moved to a fresh, sterile 1.5 ml microtube, and 50 μl of elution buffer was added. The mixture was incubated at room temperature for 2 min, then centrifuged at 13,000^x^g for 1 min to elute the DNA. After elution, the purification column was discarded, and the microtubes containing the extracted DNA were stored at −20 °C.

**Genomic DNA electrophoresis**

The agarose gel concentration used was 1 %, and it was prepared by adding 0.5 g of agarose powder to a Schott bottle. Then, 50 ml of TBE buffer was added. The bottle was heated over medium heat for about 3 min until the agarose had completely dissolved. To visualize the DNA, 5 μl of ethidium bromide was introduced and mixed thoroughly. Gel trays and combs were set up according to the number of samples. The agarose solution was poured into the gel tray and allowed to solidify. For electrophoresis, the sample cocktail composition was created with a total volume of 10 μl, containing 2 μl of genomic DNA, 1 μl of 10x BPB, and 7 μl of 1x TE buffer. A similar cocktail composition was prepared for the λ DNA marker (50 ng/μl). Once the agarose gel solidified, the comb was removed, and the gel tray was detached from the mold. The electrophoresis apparatus was set up by connecting the chamber to the power supply. The gel tray was placed in the electrophoresis chamber, and TBE buffer was added until the gel was fully submerged. DNA samples were carefully loaded into individual wells, starting with the λ DNA marker in the leftmost well, followed by the genomic DNA samples. The electrophoresis was conducted at 100 V for 30 min. Once the electrophoresis was complete, the gel was visualized and documented using a Gel Documentation System equipped with a UV transilluminator (Biometra, Germany).

**PCR amplification of the 16*Sr*RNA gene**

To prepare the PCR mixture, a sterile 0.2 ml microtube was used. The components added to the microtube were: 25 μl of KOD One Blue Mastermix, 2 μl of Primer 16SrRNA_27F (10 ng/μl), 2 μl of Primer 16SrRNA_1525R (10 ng/μl), 2 μl of the bacterial genomic DNA (10 ng/μl), and 19 μl of nuclease-free water. The PCR mixture was gently mixed and then placed in a PCR machine with settings that were optimized for the 16SrRNA primer. Following the PCR amplification, the samples were subjected to electrophoresis on a 1 % agarose gel. For this, 2 μl of a 1 kb marker was loaded into the leftmost well, followed by 5 μl aliquots of each PCR reaction mixture loaded into the subsequent wells. The electrophoresis was conducted at 100 V for 30 min to achieve proper separation of the DNA fragments. Once complete, the agarose gel was visualized and documented using a gel documentation system. Afterward, the remaining PCR reaction mixtures in the microtubes were stored at −20 °C for any future analyses or applications.

**Sequencing, bioinformatics analysis and phylogenetic tree construction**

To determine the nucleotide sequence of the 16SrRNA gene in bacterial samples, Sanger Sequencing was employed to analyze the PCR products from both the forward and reverse strands. To obtain accurate and reliable 16SrRNA gene sequences, chromatograms generated from both sequencing directions for each sample were carefully edited and verified using SeqManTM software. The resulting base sequences were then subjected to a BLAST search against the NCBI database (https://ncbi.nih.gov) to identify closely related sequences. From the BLAST results, 15 bacterial 16SrRNA gene sequences were selected from the gene bank. These selected sequences were subsequently aligned, used to construct phylogenetic trees, and analyzed for genetic distances using the MEGA X program (Vijayaraghavan et al., 2013). The Clustal W algorithm was employed for the alignment, and phylogenetic trees were generated through the Neighbor-Joining method, as outlined by Kumar et al. (2018). To evaluate evolutionary distances, the analysis was conducted using Kimura's 2-parameter method, following the approach outlined by Saitou and Nei (1987), and the original Kimura 2-parameter method proposed by Kimura (1980). The bootstrap technique was applied with a value of 1,000 to enhance the robustness of the phylogenetic trees, as introduced by Felsenstein (1987). The Pairwise Distances method was used to calculate genetic distances.

## 3 Results

### 3.1 Screening of phytase producing bacteria

A total of 3 isolates were acquired from acidification of *Thitonia diversifolia* plants in media containing Na-phytate. These isolates were labeled R5, R7, and R8, each producing distinct clear zones. The screening results for phytase-producing bacteria are shown in Table A.1 and phytase clear zone are presented in Table A.2. Isolate R8 has the largest clear zone (31.7 mm), while isolate R7 has a clear zone of 16.1 mm, and isolate R5 has the smallest clear zone (20.3 mm).

### 3.2 Enzyme activities

Enzyme activity tests revealed that isolate R5 had the highest phytase activity with 6.54 U/ml. Isolate R8 exhibited the greatest cellulase activity at 2.92 U/ml, and isolate R7 demonstrated the highest protease activity, reaching 23.4 U/ml (as depicted in Table A.3).

### 3.3 Morphological characteristics of phytase-producing bacteria

The results of the characterisation test are presented in Table A.4. All isolates had a cream color, were aerobic and classified as gram-positive bacteria. All bacterial isolates showed negative results for gas, H_2_S, indole, OF and nitrate tests. This isolate does not use glucose and sucrose but uses lactose. All isolates were negative for the mannitol test and positive for the mortality, urea, citrate, VP and gelatin tests. Isolate R5 was found to have positive results on the MR test, while isolates R7 and R8 showed negative reactions.

### 3.4 Identification of isolates using gen 16SrRNA

**Fig. A.1:**
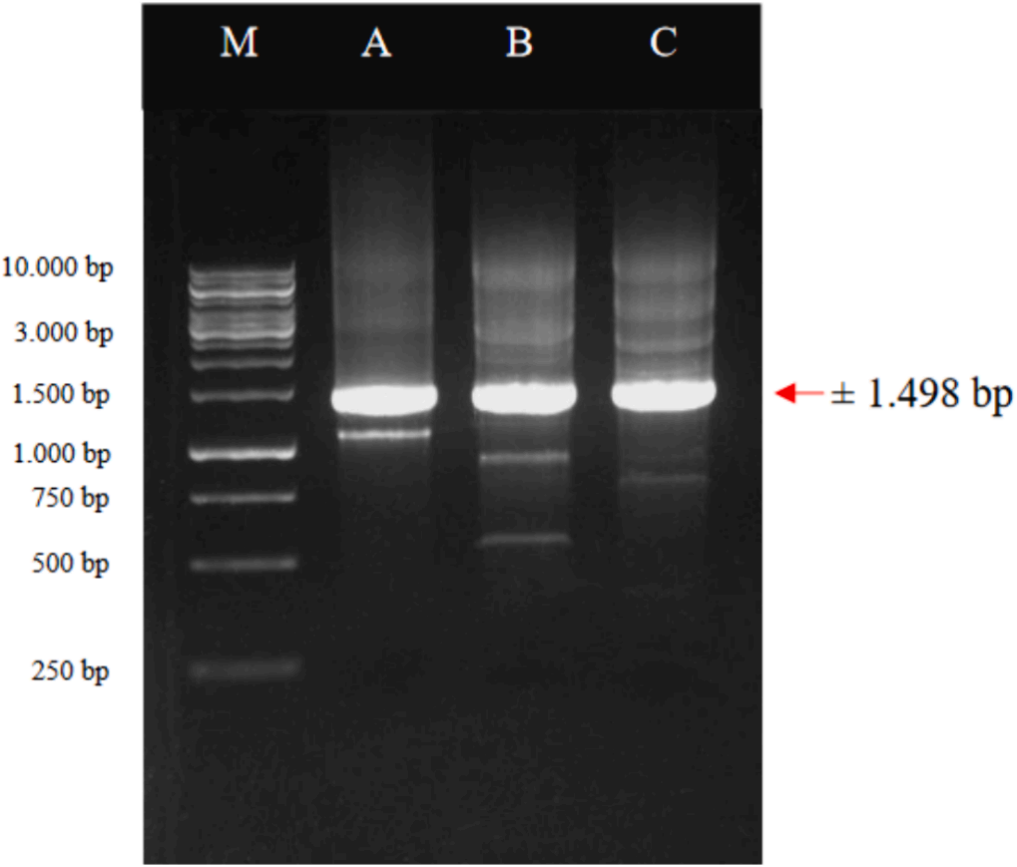
Polymerase Chain Reaction Amplification of 16S rRNA Gene from Phytate-Producing Bacteria; M (Marker 1 kb); A = Isolate R5; B = Isolate R7.

**Fig. A.2:**
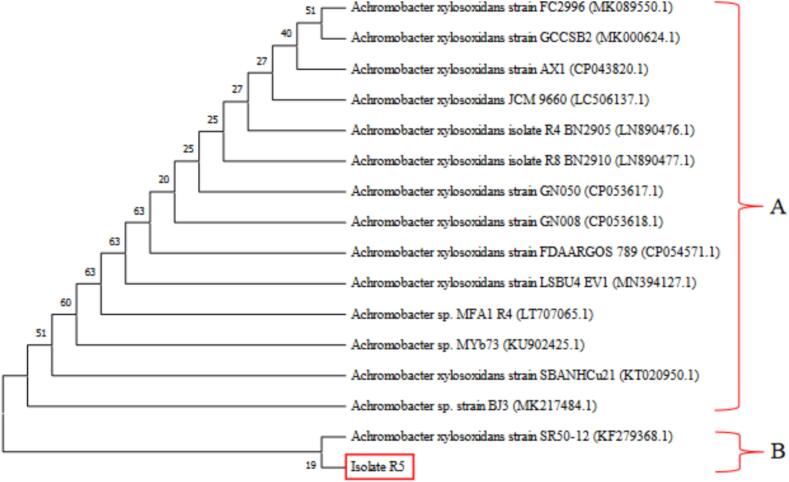
Phylogenetic Tree of 16S rRNA Gene of Phytase Producer Bacteria (Isolate 5) with Bootstrap 1000x.

**Fig. A.3:**
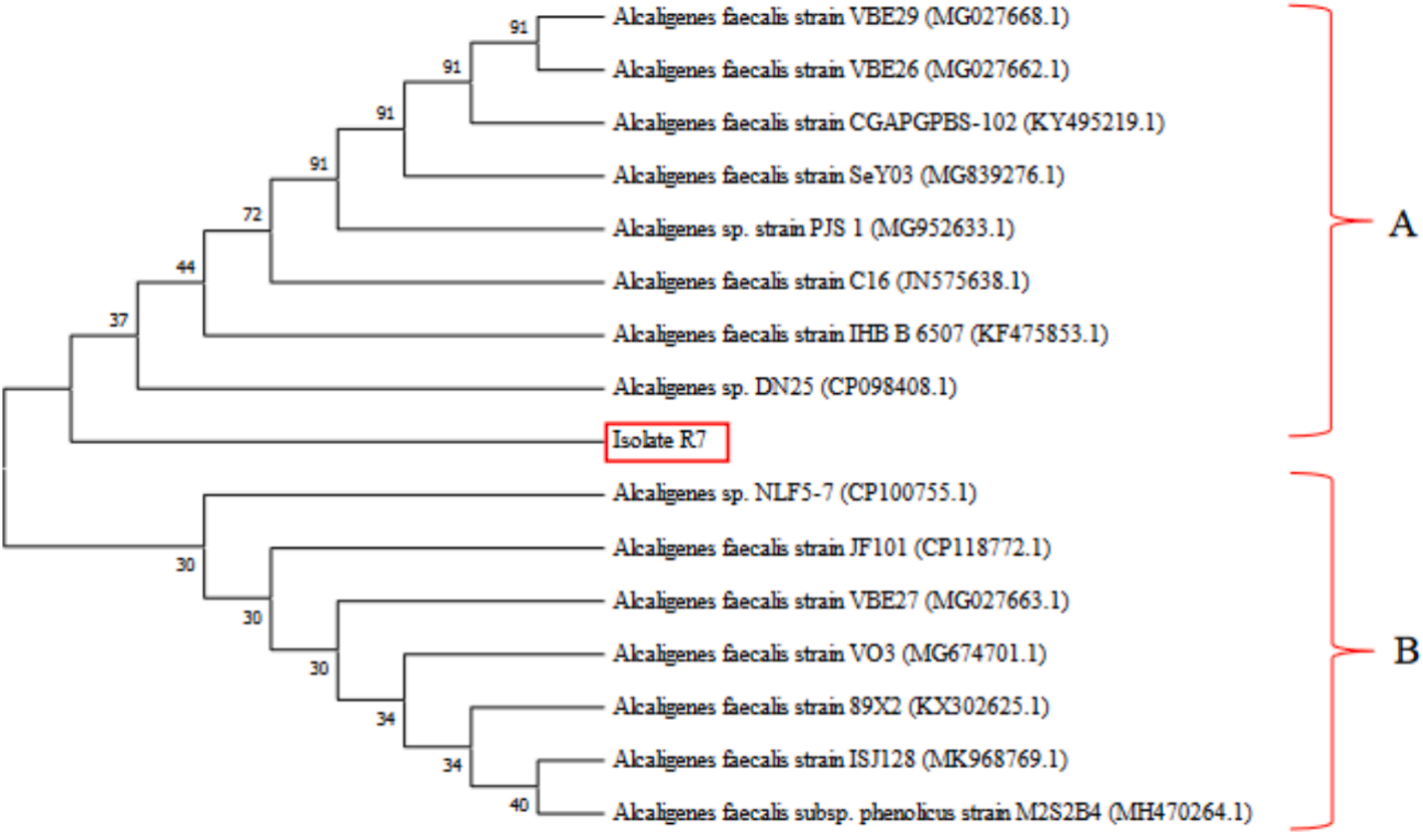
Phylogenetic Tree of 16S rRNA Gene of Phytase Producer Bacteria (Isolate 7) with Bootstrap 1000x.

**Fig. A.4:**
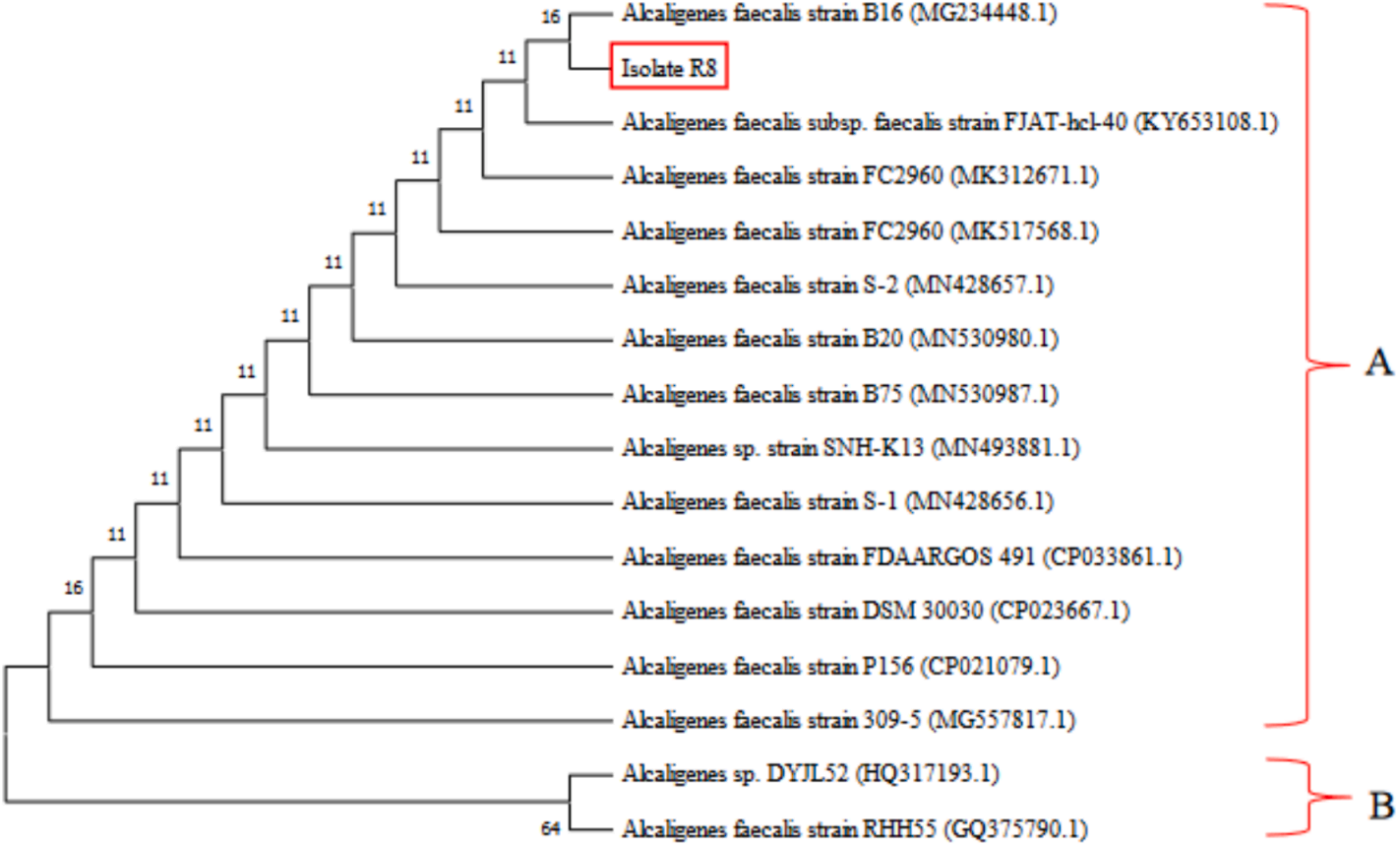
Phylogenetic Tree of 16S rRNA Gene of Phytase Producer Bacteria (Isolate 8) with Bootstrap 1000x.

**Fig. A.5:**
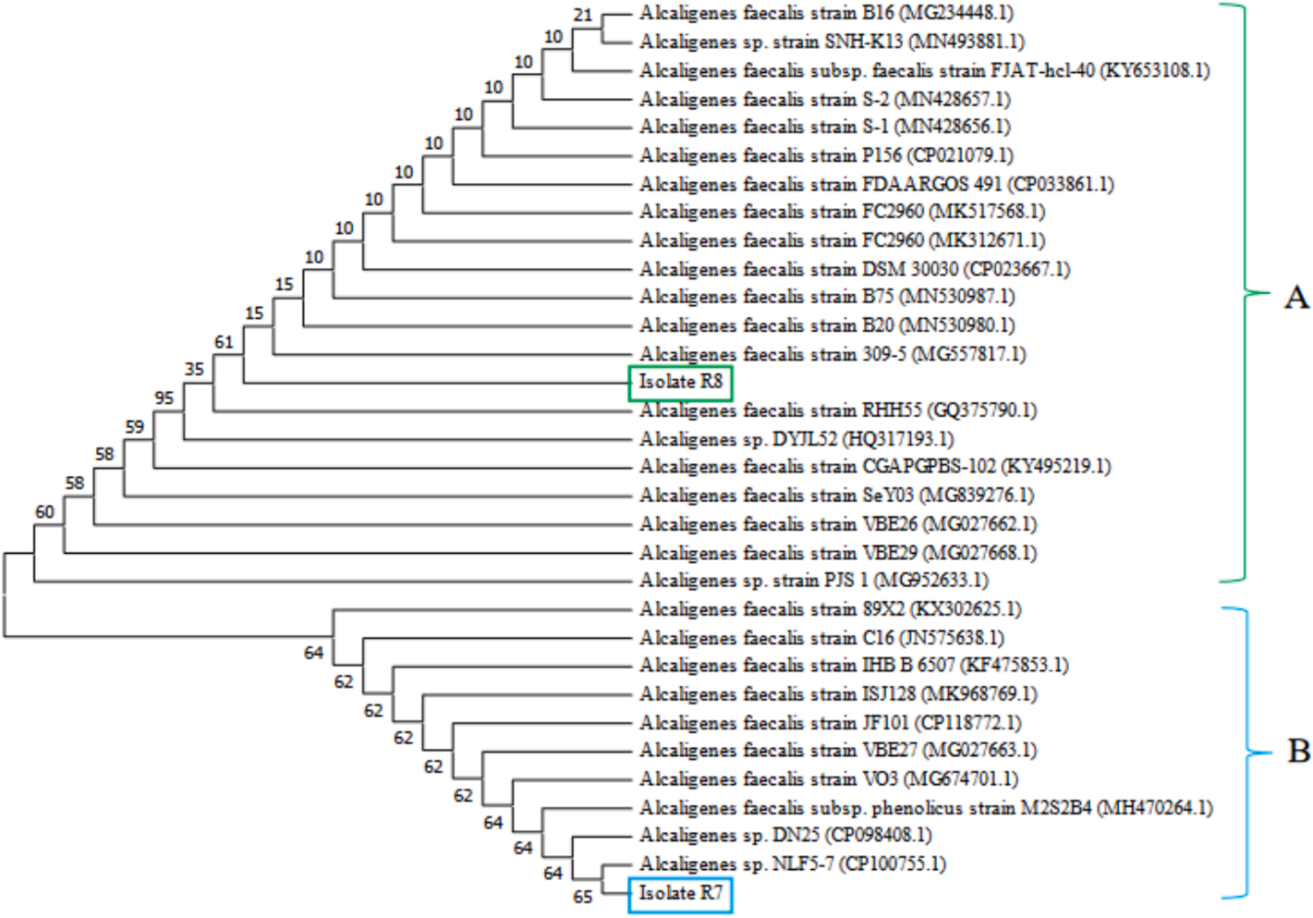
Phylogenetic Construction of Isolates R7 and R8.

Based on identification test using 16SrRNA, it was found that isolate R5 belonged to *Achromobacter xylosoxidans*, with 99.88 % similarity. Isolate R7 was identified as *Alcaligenes faecalis,* also with 99.79 % similarity, and isolate R8 shared the same identificationas as *Alcaligenes faecalis,* with 99.79 % similarity (Table A.5). The result of PCR amplification are illustrated in Fig. A.1. The phylogenetic tree of R5, R7 and R8 were presented in Fig. A.2, Fig. A.3 and Fig. A.4 respectively. The phylogenetic construction of R5 and R7 isolates can be found in Fig. A.5.

## 4 Discussion

### 4.1 Isolation and characterization of phytase producing bacteria

Semiquantitative analysis of phytase-producing bacterial activity was done by measuring the clear zone around the colony. This study identified three bacterial isolates capable of producing the phytase enzyme: R5, R7, and R8 (as shown in Table A.1). The formation of clear zones on phytase-producing media indicates that the bacteria acquired produce phytase enzyme. This is supported by Sharma and Shukla (2020) who said that the clear zones around the colony are indicative of phytase production by respective isolated bacteria.

Isolate R8 was found to have the largest phytase clear zone (31.7 mm) (Table A.2). This shows that isolate R8 has a greater capacity to produce phytase enzyme compared to other isolates. According to Onawola et al. (2019), the formation of a clear zone or holo by bacterial isolates on a phytase-specific medium deomonstrates the ability to produce phytase, the enzyme required for the solubilization of phytate.

### 4.2 Phytase, cellulase and protease activity

The results of pH measurements and the enzymatic activities of phytase, cellulase, and protease in each isolate are detailed in Table A.3. The phytase enzyme activities for isolates R5, R7, and R8 were 6.54 U/ml, 5.45 U/ml, and 5.53 U/ml, respectively. Isolate R5 demonstrated the highest phytase activity among the three and was identified as *Alcaligenes faecalis* (Fig. A.2)*.* According to Vijayaraghavan et al. (2013), phytase from *Alcaligenes* sp. was able to release inorganic phosphate from plant phytate by 1740 and 1655 mg of inorganic phosphate per kg of plant phytate from corn and chick feed.

The procedure for evaluating phytate value derived from the phytase enzyme activity in *Alcaligenes faecalis* remains uncertain due to inconsistent timing in the testing process. Phytase-producing bacteria, which typically generate substantial quantities of phytic acid, can experience a drop in activity when measured at varying time intervals. This issue was noted in the study by Pazla et al., (2023), where *Lactobacillus bulgaricus* within *Mirasolia diversifolia* exhibited a significant reduction in phytic acid content from 21.14 U/ml after one day of fermentation to 5.26 U/ml. Additionally, it was observed that introducing a 3 % concentration of *L. bulgaricus* to the fermentation of *M. diversifolia* over a period of five days led to a minimum phytic acid content of 4.30 mg/100 g, indicating a degradation rate of 63.62 %.

The phytase enzyme is capable of breaking down phytate, a complex organic compound containing phosphate. Phytase enzyme activity releases inorganic phosphate and inositol from phytate. In addition to its primary role in increasing phosphate and nutrient availability, phytase enzymes can also influence feed palatability (Humer and Zebeli, 2015).

The results of pH measurements in the growth media of the isolates showed values ranging from 8.62 to 9.23. Phytase enzyme production was observed to be highest in isolate R5, where the growth media had a pH of 8.62. As the pH level increased, reaching 9.0, the production of the phytase enzyme decreased. This decrease in enzyme production with elevated pH levels can be attributed to the diminished metabolic activity of bacteria under such conditions, resulting in lower enzyme yields (Lan et al., 2002).

Phytic acid is efficiently broken down by phytase enzymes produced by various microbes at a pH of 5.0. The activity of phytases depends upon the pH and temperature. Compared to plant phytases, microorganism-produced phytases have a higher pH and thermostability (Azeem et al., 2014). However, each microorganism and its species has a different optimal temperature and pH level. However, ruminants have a natural ability to absorb phytate because microbes in the rumen can produce their own phytase enzymes. In animal feed, the addition of bran or other supplements, such as those derived from *Tithonia*, can contribute additional nutrients. Thus, the use of *Achromobacter* and *Alcaligenes* species to hydrolyze phytic acid in *Tithonia diversifolia* for use as feed components remains feasible under specific conditions (De Boland et al., 1975, Kaswarjono et al., 2016).

*Achromobacter* is not categorized as a phytase-producing bacterium, like *Bacillus sp*., because its phytase activity is relatively low, at 8.84 U/ml (Bikash et al., 2017). Despite this, the bacterium shows potential for phytase production based on its processing capabilities. This observation aligns with experimental results from isolate R5, which demonstrated a phytase activity of 6.54 U/ml. Further validation can be achieved by testing it on suitable substrates. The presence of phytase-producing bacteria during the acidification process of *Tithonia diversifolia* offers a promising alternative source for ruminant feed.

The cellulase activity of isolates R5, R7, and R8 was measured at 2.60, 2.87, and 2.92 U/ml, respectively. Cellulase activity correlates with the production of reducing sugars; higher cellulase enzyme activity typically results in greater production of reducing sugars. The observed differences in enzyme activity across the isolates can be linked to the unique strains of bacteria employed. Bacteria from different strains often demonstrate varying enzyme activities. Some microbes show high enzyme activity, while others exhibit lower levels (Yi et al., 2019). Consequently, the choice of bacterial species plays a crucial role in determining enzyme activity. *Alcaligenes faecalis*, for example, is known as a source of *β-glucosidase* or *cellobiase*, enzymes responsible for hydrolyzing cellobiose into glucose monomer units (Han and Srinivasan, 1969).

The protease enzyme activities of isolates R5, R7, and R8 were 22.3, 23.4, and 22.2 U/ml, respectively. These values are considerably lower than those reported by Han and Srinivasan (1969), who achieved 115.9 U/ml at pH 9 in both a sodium acetate buffer and a phosphate buffer through the use of enzymes derived from *Alcaligenes*. Similarly, Marathe et al. (2018) discovered that alkaline proteases extracted from Bacillus bacteria displayed higher enzyme activity, reaching 103.26 ± 2.11 U/ml at pH 10 with a sodium acetate buffer and 92.25 ± 1.82 U/ml at pH 10 with a phosphate buffer. By comparison, the enzyme isolated from *Alcaligenes* exhibited the most significant enzyme activity at 96.12 ± 1.59 U/ml at pH 9 in a sodium acetate buffer and 89.5 ± 1.89 U/ml at pH 9 with a phosphate buffer. Protease enzyme activity from *Pseudomonas* also achieved high levels, with activity reaching 90.34 ± 1.09 U/ml at pH 9 with a sodium acetate buffer and 85.4 ± 1.82 U/ml at pH 9 with a phosphate buffer. The reduced activity of protease enzymes isolated from *Alcaligenes sp*. in this study may be attributed to their cultivation on a general, non-selective medium. Thus, when nutrients are supplemented with specific buffer solutions or sodium, there is a potential to increase protease enzyme activity.

### 4.3 Morphology characteristic of isolates

The morphological characteristics of each isolate are detailed in Table A.4. According to the research findings, all bacterial colonies were cream-colored. All isolates are gram-positive bacteria, distinguished from gram-negative bacteria by their lack of an outer membrane and the presence of a thick peptidoglycan layer surrounding the plasma membrane, which helps protect them from harsh environmental conditions (Jubeh et al., 2020). Isolates R5, R7, and R8 are aerobic bacteria, meaning they require oxygen for survival and growth (Srifani et al., 2023).

The Voges-Proskauer (VP) test is a biochemical assay designed to detect the capability of specific bacteria to metabolize glucose into an intermediate neutral product called acetoin during fermentation. To test for acetoin, certain reagents are added, and a positive result is indicated by a red or pink coloration. In this study, all isolates tested positive for the VP test, demonstrating that they can produce acetyl methyl carbinol, also known as acetoin (Srifani et al., 2023).

The methyl red (MR) test is a biochemical assay used to determine if microorganisms can produce and maintain an acidic final product through the oxidation of glucose. Positive results are indicated by a change in the color of the top of the media to red after 3–5 drops of a 1 % methyl red reagent are added (Puspadewi et al., 2017). In this study, isolate R5 displayed a positive reaction to the MR test, while isolates R7 and R8 showed negative reactions.

The Indole test is designed to determine whether bacteria possess the enzyme tryptophanase, enabling them to oxidize the amino acid tryptophan into indole (Rahman et al., 2019). To conduct this test, a specific reagent is added to the bacterial culture, and a positive result is indicated by the development of a red color. In this study, none of the isolates tested positive for the Indole test, indicating that they do not have the tryptophanase enzyme.

The H_2_S test is used to detect the formation of hydrogen sulfide gas by certain bacteria. A positive result is indicated by the appearance of a black precipitate, while a negative result shows no such formation. In this study, all isolates were negative for the H_2_S test, indicating that they do not produce hydrogen sulfide. The citrate test aims to assess the ability of bacteria to use citrate as a carbon and energy source (Yaqoob et al., 2022). A positive result is indicated by a color change to blue, whereas a negative result keeps the media green. All isolates in this study showed a positive result on the citrate test, suggesting that they can metabolize citrate.

The glucose, maltose, sucrose, mannitol test aims to determine the ability of microorganisms to degrade and ferment carbohydrates by producing acid and gas (Sulistijowati et al., 2021). In this test, the ability to ferment different sugars, such as glucose, maltose, sucrose, and mannitol, is examined. All isolates in this study were shown to be unable to ferment these carbohydrates. Additionally, a lactose fermentation test was conducted to evaluate if the isolates could ferment lactose. The results indicated that all isolates showed a positive reaction, suggesting that they have the ability to ferment lactose.

The urea test aims to determine bacteria that have the urease enzyme (Omoregie et al., 2019). Certain bacteria can hydrolyze urea and form ammonia, causing a red color due to the phenol red indicator. The formation of ammonia causes the pH value to become alkaline so that if the urea test shows a pink color in the media, it means the test is positive. All isolates in this study have ability to produce urease enzyme.

The motility test is a test that aims to confirm whether the microorganisms found belong to the group of bacteria that have a flagellum as a means of movement. Positive results were proven by the presence of spreading colony growth and the media becoming cloudy like fog after 1 bacterial isolate was implanted perpendicularly in the media (Rahmawati and Isnaeni, 2016). All isolates in this study showed positive reaction to motility test.

The gelatin test is a method used to identify bacteria capable of hydrolyzing gelatin into simpler compounds, including amino acids (Raharja et al., 2022). In this procedure, bacterial isolates are inoculated into a gelatin-based medium to observe their ability to produce gelatinase, an enzyme responsible for hydrolyzing gelatin. A positive result is indicated by the liquefaction of the medium in the test tube, suggesting that the bacteria have hydrolyzed the gelatin. Conversely, a negative result is shown by the medium remaining solid or freezing. All isolates in this study demonstrated positive gelatinase activity, indicating that they can hydrolyze gelatin into simpler compounds.

### 4.4 16*Sr*RNA gene analysis of phytase producing bacteria

Based on the implication results, the sample DNA isolates are shown in Fig. A.1. The results obtained revealed parallel DNA bands at the 1498 bp marker. All three isolates successfully amplified the 16*Sr*RNA gene, resulting in the expected DNA fragment size of 1500 bp.

***Isolate R5***

From the three BLAST sequence data of the forward primer reading results of Isolate R5 bacteria, the query cover value and percent identity for *Achromobacter xylosoxidans* strain 23BA2-LCU-ID-02 were 96 % and 77.27 %, respectively. For the uncultured bacterium clone ncd175a01c1 and clone ncd75b12c1, the query cover values were 11 % and 8 %, respectively. Additionally, the percent identity values for each clone were 81.52 % and 86.76 %, respectively. However, due to the limited number of three BLAST results obtained, the 16*Sr*RNA gene fragment constituent sequences from this forward primer reading were not further analyzed through bioinformatics methods.

Out of 100 sequence data obtained from BLAST results of the R5 bacterial isolate, the query cover values ranged from 99 % to 100 %. The percent identity values were between 99.63 % and 99.88 %. To further analyze genetic kinship, the top 15 sequence data sets of *Achromobacter* bacteria from the GenBank database were selected for subsequent evaluation.

The BLAST results provided taxonomic information for the 100 selected sequence data, revealing that the R5 bacterial isolate belongs to the genus *Achromobacter*. Among the 100 selected sequences, 49 were identified as *Achromobacter xylosoxidans*, indicating that nearly half of the analyzed data confirmed this specific bacterial species.

MEGA X software (Fig. A.2) was utilized for constructing phylogenetic trees and calculating genetic distances. The Kimura 2-parameter method was applied for the genetic distance analysis. The results from this analysis, comparing Isolate R5 with 15 other bacteria, are shown in the figure below. The five bacteria with the closest genetic distance to Isolate R5 had a value of 0.001224. These bacteria are identified as *Achromobacter xylosoxidans strain LSBU4 EV1, Achromobacter* sp. *MFA1 R4, Achromobacter* sp. *MYb73, Achromobacter xylosoxidans strain SBANHCu21, and Achromobacter xylosoxidans strain SR50-12.*

Based on the phylogenetic tree construction, bacterial grouping was observed to be divided into two main branches. The first cluster, known as cluster A, consisted of 14 bacteria, while cluster B contained 2 bacteria. The position of Isolate R5 bacteria, indicated by a red box, is located within cluster B. In the nearest branch, Isolate R5 bacteria are closely related to *Achromobacter xylosoxidans* strain SR50-12 (**KF279368.1**). The results from BLAST, sequence alignment, genetic distance calculation, and phylogenetic tree construction with 16S rRNA gene sequences all indicated that Isolate R5 belongs to the species *Achromobacter xylosoxidans*.

*Achromobacter* has the capability to hydrolyze phytate or produce phytase when provided with an appropriate substrate or a modified growth medium. In a study where bran was used as the substrate, phytase production increased by 11.2 times, achieving a concentration of 8.84 U/ml. This significant increase in enzyme activity suggests that the phytase produced by *Achromobacter* is not only stable but also potentially ideal for use as a feed supplement.

***Isolate R7***

BLAST results provide taxonomic information for the 100 selected sequence data. Based on these results, it was determined that bacterial isolate R7 belongs to the genus *Alcaligenes*. Among the 100 sequence data from the BLAST results, 57 were identified as *Alcaligenes faecalis*. The query cover value for these BLAST results of bacterial isolate R7 was 100 %. The percent identity values acquired ranged from 99.57 % to 99.79 %. A set of 15 *Alcaligenes* bacterial sequence data from the GenBank were chosen for further use in genetic kinship analysis. The phylogenetic tree in Fig. A.3 illustrates the relationship between bacterial isolate R7 and 15 comparative bacteria sourced from GenBank, determined using 16S rRNA gene fragment sequences.

Based on the phylogenetic tree construction (Fig. A.3), the bacterial grouping is divided into two main branches, resulting in two clusters. The first cluster, denoted as cluster A, comprises 9 bacteria, while cluster B consists of 7 bacteria. Bacterial Isolate R7, indicated by the red box, is placed in cluster A. The members of cluster A from the GeneBank include 6 *Alcaligenes faecalis* bacteria and 2 *Alcaligenes* sp. Based on the results from BLAST, alignment, genetic distance calculations, and phylogenetic tree construction, it can be concluded that bacterial Isolate R7 is a species of *Alcaligenes faecalis* bacteria.

***Isolate R8***

Taxonomically, it was determined that bacterial isolate R8 belongs to the genus Alcaligenes. Among the 100 selected sequence data, 61 were identified as the bacterial species *Alcaligenes faecalis*. In the BLAST results of the 100 sequence data of Isolate R8 bacteria, a query cover value of 100 % was achieved. The percent identity value acquired fell within the range of 99.85 % to 100 %. A total of 15 *Alcaligenes* bacterial sequence data from the GeneBank were selected for further use in genetic kinship analysis. The following is the phylogenetic tree that demonstrates the relationship between Isolate R8 bacteria and 15 comparative bacteria retrieved from GeneBank, utilizing 16S rRNA gene fragment sequences.

Based on the phylogenetic tree construction (Fig. A.4), the bacterial grouping is divided into two main branches, resulting in two clusters. The first cluster, labeled as cluster A, comprises 14 bacteria. Cluster A consists of 12 bacteria of the *Alcaligenes faecalis* species and 1 bacterium of *Alcaligenes* sp. from the GeneBank. Cluster B consists of 2 bacteria, including 1 *Alcaligenes sp*. and 1 *Alcaligenes faecalis* bacterium from the GeneBank. Bacterial Isolate R8, indicated by the red box, is placed in cluster A. In the nearest branch, Isolate R8 bacteria are closely related to *Alcaligenes faecalis* strain B16 (**MG234448.1**). According to the results from BLAST, alignment, phylogenetic tree construction, and genetic distance calculations, it can be concluded that bacterial Isolate R8 is identified as a bacterium of the species *Alcaligenes faecalis*.

The phytase enzyme produced by *Alcaligenes sp*. can function optimally at pH 7.0–8.0. This property can be specifically utilized in fish feed because the pH of the fish gut falls within this range of values. However, this phytase can also be applied in other animal feeds as it effectively enhances phosphorus bioavailability (Kumar et al., 2012).

### 4.5 Phylogenetic construction of Isolate R7 and R8

Both Bacterial Isolate R7 and R8 were designated as species through the analysis of 16S rRNA gene fragment sequences. To assess the similarity between bacterial isolates R7 and R8, a phylogenetic tree was constructed. In addition to the 16S rRNA gene fragment sequences of bacterial Isolate R7 and R8, the sequence data of comparator bacteria for each isolate from the GeneBank was also included. The phylogenetic tree illustrates the relationship between bacterial Isolate R8, Isolate R7, and 30 comparative bacteria from the GeneBank based on 16S rRNA gene fragment sequences.

Based on the phylogenetic tree construction (Fig. A.5), the bacterial grouping is divided into two main branches, resulting in two clusters. The first cluster, labeled as cluster A, comprises 21 bacteria. Bacterial Isolate R8 (indicated by the green box) is placed in cluster A along with its comparator bacteria and some of the comparator bacteria from Isolate R7. The second cluster, labeled as cluster B, comprises 11 bacteria. Isolate R7 bacteria (indicated by the blue box) are placed in cluster B along with 10 comparator bacteria from Isolate R7 in the GeneBank. From this phylogenetic tree construction, it is confirmed that bacterial Isolate R7 and Isolate R8 are identified as *Alcaligenes faecalis* bacteria.

## 5 Conclusion

Based on the series of research conducted, three isolates of phytase-producing bacteria were identified: R5, R7, and R8. Isolates R5, R7 and R8 produced phytase clear zones of 20.3 mm, 16.1 mm, 31.7 mm, and they had phytase activity of 6.54 U/ml, 5.45 U/ml, and 5.53 U/ml, respectively. Isolate R5 belongs to the bacterial species *Achromobacter xylosoxidans,* while isolates R7 and R8 belong to *Alcaligenes faecalis*. Further experimentation is needed to evaluate their phytate degradation capacity, which could potentially enhance the palatability and digestibility of ruminant feed.

## CRediT authorship contribution statement

**Roni Pazla:** Supervision. **Gusri Yanti:** Writing – original draft. **Novirman Jamarun:** Conceptualization. **Mardiati Zain:** Conceptualization. **Hera Dwi Triani:** Formal analysis, Methodology. **Ezi Masdia Putri:** Methodology, Project administration. **Anifah Srifani:** Data curation, Validation.

## Declaration of competing interest

The authors declare that they have no known competing financial interests or personal relationships that could have appeared to influence the work reported in this paper.

## Figures and Tables

**Table A.1:** Results of Screening for Phytase-Producing Bacteria from Acidified *Tithonia diversifolia.*

Parameter	Isolate		
	R5	R7	R8
Clear zone			

**Table A.2:** Phytase clear zone of isolates.

Isolat	Clear zone (mm)
R5	20.3 mm
R7	16.1 mm
78	31.7 mm

**Table A.3:** Phytase, Cellulase, and Protease Enzyme Activities of Isolates from *Thitonia diversifolia* Acidification (n = 3).

Parameter	Isolat		
	R5	R7	R8
pH	8.62 ± 0.12	9.23 ± 0.22	9.18 ± 0.20
Phytase (U/ml)	6.54 ± 0.32	5.45 ± 0.29	5.53 ± 0.19
Cellulase (U/ml)	2.60 ± 0.66	2.87 ± 0.76	2.92 ± 0.72
Protease (U/ml)	22.3 ± 0.67	23.4 ± 0.54	22.2 ± 0.62

**Table A.4:** Traits of Bacterial Isolates with Phytase-Producing Abilities.

Characteristics	Isolate		
	R5	R7	R8
Blood Agar	+	+	+
Colonies (colour)	Cream	Cream	Cream
Gram	+	+	+
Aerob/Anaerob	A	A	A
TSIA	MK	MK	MK
Gas	−	−	−
H2S	−	−	−
Mortilitas	+	+	+
Indole	−	−	−
Urea	+	+	+
Citrat	+	+	+
Lactose	+	+	+
Glucose	−	−	−
Sucrose	−	−	−
Mannitol	−	−	−
MR	+	−	−
VP	+	+	+
OF	−	−	−
Nitrat	−	−	−
Gelatin	+	+	+

**Table A.5:** Similarity of Isolates with GenBank.

Isolat	GenBank	% Similirity	Accesion number
R5	*Achromobacter xylosoxidans*	99.88	MN394127.1
R7	*Alcaligenes faecalis*	99.79	MK968769.1
R8	*Alcaligenes faecalis*	100	MN530987.1

## Appendix A

Fig. A.1, Fig. A.2, Fig. A.3, Fig. A.4, Fig. A.5, Table A.1, Table A.2, Table A.3, Table A.4, Table A.5
